# Sarcoma Common MHC-I Haplotype Restricts Tumor-Specific CD8+ T Cell Response

**DOI:** 10.3390/cancers14143414

**Published:** 2022-07-14

**Authors:** Laura Mosca, Alessandra de Angelis, Andrea Ronchi, Annarosaria De Chiara, Flavio Fazioli, Carlo Ruosi, Lucia Altucci, Mariarosaria Conte, Filomena de Nigris

**Affiliations:** 1 Department of Precision Medicine, School of Medicine, University of Campania “Luigi Vanvitelli”, 80138 Naples, Italy; Laura.Mosca@unicampania.it (L.M.); alessandra.dea@gmail.com (A.d.A.); Lucia.Altucci@unicampania.it (L.A.); Mariarosaria.conte@unicampania.it (M.C.); 2 Pathology Unit, Department of Mental and Physical Health and Preventive Medicine, University of Campania “Luigi Vanvitelli”, 80138 Naples, Italy; andrea.ronchi@gmail.com; 3 Division of Anatomy, Istituto Nazionale Tumori IRCCS—Fondazione G. Pascale, 80131 Naples, Italy; annarosaria.dechiara@gmail.com; 4 Division of Skeletal Muscle Oncology Surgery, Istituto Nazionale Tumori IRCCS—Fondazione G. Pascale, 80131 Naples, Italy; flavio.fazioli@gmail.com; 5 Department of Public Health, School of Medicine, University Federico II, 80131 Naples, Italy; carlo.ruosi@gmail.com; 6 BIOGEM, Molecular Biology and Genetics Research Institute, 83031 Ariano Irpino, Italy

**Keywords:** immunotherapy, CD8 T cells, PD-L1, HLA

## Abstract

**Simple Summary:**

Immunotherapy targeting immune checkpoint pathways have recently attracted great attention in cancer treatment, but better strategies are needed to identify patients likely to benefit from it. The major histocompatibility complex (MHC) class I expression in cancer cells greatly influences the outcome of T cell-mediated immunotherapy. Here, we determined the prevalent HLA class I allelic variants in a sarcoma population. We characterized patient CD8+ T-cells and demonstrated low cytolysis to autologous tumor cells. Moreover, we used a co-culture model of autologous T-cells and PD-L1-deficient or positive biopsies of rare sarcomas to determine whether HLA-I influences tumor survival.

**Abstract:**

The major histocompatibility complex (MHC) class I expression in cancer cells has a crucial impact on the outcome of T cell-mediated cancer immunotherapy. We now determined the HLA class I allelic variants and their expression in PD-L1-deficient and positive rare sarcoma tissues. Tumor tissues were HLA-I classified based on HLA-A and -B alleles, and for class II, the HLA-DR-B by Taqman genomic PCRs. The HLA-A24*:10-B73*:01 haplotype was the most common. A general down-regulation or deletion of HLA-B mRNA and HLA-A was observed, compared to HLA-DR-B. HLA-I was almost too low to be detectable by immunohistochemistry and 32% of grade III cases were positive to PD-L1. Functional cytotoxic assays co-culturing patient biopsies with autologous T cells were used to assess their ability to kill matched tumor cells. These results establish that deletion of HLA-I loci together with their down-regulation in individual patient restrict the autologous lymphocyte cytotoxic activity, even in the presence of the immune checkpoint blocking antibody, Nivolumab. Additionally, the proposed cytotoxic test suggests a strategy to assess the sensitivity of tumor cells to T cell-mediated attack at the level of the individual patient.

## 1. Introduction

Soft tissue sarcoma (STS) is a heterogeneous group of mesenchymal tumors with distinct histology and clinical behavior [1,2,3]. Traditional treatment of patients with advanced stages of diseases is based on surgery, systemic chemotherapy and radiation, but 40% of cases will develop metastases or recurrences within 2 years [3]. Novel therapeutic approaches have been identified with new clinical management prospectives, including immune checkpoint inhibitors [4,5,6]. PD-L1/PD-1 inhibitors are the most investigated immunotherapeutic and have shown their efficacy in various cancers [7,8,9,10] and in patient specific three-dimensional preclinical models of rare sarcomas [11,12,13]. In advanced sarcoma trials, a generally low response rate was reported. SARC028 is one of the first phase II trials in which the patients received the anti-PD-1 antibody pembrolizumab as monotherapy. Data indicated that the response rate was associated with specific tumor histology. In particular, undifferentiated pleomorphic sarcoma (UPS) and dedifferentiated liposarcoma (DDLPS) subtypes were the best responders to treatment. However, the total rate of response was only 18% [14], generally associated with higher densities of cytotoxic T cell infiltration in the tumors of responders [14]. Similarly, a multicenter randomized phase II clinical trial (Alliance A091401) using Nivolumab as monotherapy or in combination with Ipilimumab reported limited efficacy in an unselected sarcoma population [15]. Subsequent clinical trials testing immunotherapeutic alone or in combination with other agents correlate immune-related pathways with improved efficacy in sarcoma subtypes [16,17]. Interestingly, to date, the objective response rate to pembrolizumab observed in SARC028 remains the highest among studies evaluating immune checkpoint therapy in similar patient cohorts.

The search for more effective immunotherapies is rendered more difficult by our limited knowledge of the complex interplay between the tumor type and the immune system, and by the fact that many patients develop resistance to treatment, which may also be due heterogeneity of immune mechanisms. A better understanding of these interactions may not only lead to more effective treatment, but also to the identification of more predictive biomarkers to treatment response of STS [18]. Evidence indicates that rearrangements (duplication) in the 6p21 chromosomal region are important in the pathogenesis and prognosis of osteosarcoma and an anomaly of the 6p21 region resulting in a rearrangement of HMGA1 was reported to be the second most frequent in adult lipoma [19,20]. The major histocompatibility complex (MHC) is composed of two classes of human leukocyte antigen genes (HLA), which map on the 6p21 chromosomal region and play a critical role in immune responses to pathogens and cancers [21]. HLA class I molecules (categorized into HLA-A, -B, and -C according to their gene locus) are expressed on all nucleated cells and present antigens to CD8+ T lymphocytes. In turn, CD8+ T cells require CD4+ T helper cells activated via HLA class II molecules (categorized into HLA-DR, -DQ, and -DP loci) [22,23]. Selective loss or genomic alteration of HLA class I haplotypes, together with the co-option of immune checkpoint PD-1/PD-L1, have been described in cancer as an escape mechanism to host immune defenses [24,25,26,27]. Evidence suggests that reduced HLA class I expression is associated with high PD-L1 positive immunostaining [28,29,30,31]. Clinical studies in several cancer types confirmed that patients responded poorly to immunotherapy treatment when tumors showed low expression levels of HLA class I molecules [31,32]. In sarcoma tissues, limited information is available about HLA-I immunological molecules and their correlation to immune-checkpoint immunotherapy.

The present study investigated whether the HLA-I haplotype and its expression in low- and high-grade sarcoma tissues influences the activity of cytotoxic T cells. For this purpose, we analyzed HLA class I antigens in frozen sarcoma tissues, and genotyped HLA-A, -B alleles and HLA-DR by multiplex Taqman. We also determined the prevalence of the HLA haplotype and investigated the functional relationship with PD-L1 by a CD8+ T cells cytotoxicity assay.

## 2. Materials and Methods

### 2.1. Patients and Cell Cultures

Tissues and blood were collected during surgery from patients (*n* = 40) who received a diagnosis of soft tissue sarcoma at the Vanvitelli University and Istituto Tumori G. Pascale of Naples from 2010–2020. The tumors included different soft tissue histology, confirmed by a senior musculoskeletal pathologist according to the WHO classification system [2]. Only patients for whom immunohistochemistry data, follow-up and sufficient DNA, RNA and proteins were available were selected, independently of gender, age and ethnicity. None of these patients received any chemotherapy before surgery. Tissues and selected peripheral blood mononuclear cells (PBMCs) were frozen at −135 °C for later analysis. In addition, cell cultures were prepared from the fresh tissues of four patients operated in 2021. Tissues from sarcoma patients were digested with collagenase type IV (0.2 mg/mL, Gibco, Italy) for 1 h at 37 °C and grown in RPMI 1640 medium with 10% FBS, L-glutamine and penicillin/streptomycin (Life Technologies, Milan, Italy). Written informed consent for tissue collection and medical information used for the study and later publication was obtained from each patient at their first visit without personally identifiable information.

### 2.2. DNA/RNA Isolation and cDNA Synthesis

Aliquots of 2 mg frozen patient tissues were fragmented with TissueRuptor II (Qiagen, Milan, Italy) and resuspended in 1 mL of DNA solution from QIAamp DNA Tissue Kit (QIAGEN). DNAs were prepared following the manufacturer’s protocol. Total RNA was obtained from the same amount of tissue homogenized and suspended in TRIZOL reagent according to the manufacturer’s instructions (Sigma, Saint-Quentin, France). DNA and RNA concentration and quality were determined by measuring the A260/280 ratio with a NanoPhotometer P300 (Implen, Munich, Germany). Then, 1 µg of total RNAs were reverse-transcribed, using a reverse transcriptase kit (Invitrogen, Milan, Italy). CDNAs amplification were carried out at 95 °C for 15 min, followed by 40 cycles of three steps of denaturation at 94 °C for 15 s, annealing at 60 °C for 30 s, and extension at 72 °C for 30 s using SYBR Green PCR MasterMix (Applied Biosystems, San Cose’, CA, USA) with a Bio-Rad CFX96 PCR instrument. A melting curve analysis was performed from 70 °C to 95 °C in 0.3 °C intervals. Each measurement was performed in triplicate. GAPDH was used to normalize the RNA input. qRT-PCR primer sequences are listed in online supplemental Table S3. Relative mRNA expression was calculated with the formula 2^−ΔΔ*Ct*^.

### 2.3. HLA Genotyping

A total of 40 ng of genomic DNA per well was used for HLA genotyping by GeneFinder™HLA-ABDR Plus RealAmp Voden kit (IFMR-10-CFX), which covers HLA allele sequence matches in the polymorphic region encoding the antigen presentation domain, exon 2 (class II) or exons 2 and 3 (class I). The amplifications were carried out at 96 °C for 5 min, followed by five cycles of three steps consisting of denaturing at 96 °C for 25 s, annealing 70 °C 45 s and extension at 72 °C for 30 s, followed by 32 cycles of three steps consisting of denaturation at 96 °C for 25 s, annealing at 65 °C for 45 s and extension at 72 °C for 30 s on a Bio-Rad CFX96 PCR instrument. Interpretation of Taqman data was performed with the GENEFINDER program.

### 2.4. Western Blots

In total, 2 mg of frozen tumor tissues were fragmented with TissueRuptor II (Qiagen) in RIPA buffer. Then, 30 µg of total proteins from tumor tissues were separated by SDS-polyacrylamide gel electrophoresis and transferred to a nitrocellulose membrane. The membrane was blocked with 5% milk for 1 h at room temperature and incubated with a primary antibody at 4 °C overnight. The human-specific anti-rabbit PD-L1 was purchased from Abcam (Ab213524). Primary antibodies were used at a dilution of 1:1000, and all secondary HRP-conjugated antibodies at a dilution of 1:5000. Blots were developed using enhanced chemiluminescence detection reagents ECL (Cyanagen, Bologna, Italy) and exposed to X-ray film. Gamma Tubulin purchased from Abcam (Ab213524) was used as endogenous control in Western blots.

### 2.5. Complement-Dependent Cytotoxicity Assay

To detect complement-dependent cytotoxicity, we used 48 Terasaki multiwell plates and a modification of the standard histocompatibility/tissue typing/HLA method (One Lambda, Thermo Fisher, Waltham, CA, USA). Then, 2 µL of control positive or negative HLA-I (One Lambda) was added and incubated for 1 h at 25 °C, followed by incubation with 5 µL of complement for 2 h at 25 °C and then by 5 µL of a stop solution (ethidium bromide and acridine orange). When antibodies present in positive control serum bind HLA molecules expressed by tumor cells and fix complement, the cells undergo complement-mediated damage and death. The dish was then read using a Nikon Diaphot-TMD inverted microscope controlled by Lambda Scan automated micro fluorometer hardware (One Lambda) and the image captured by a Nikon digital camera.

### 2.6. Immunohistochemistry

Surgical tissues were formalin-fixed and embedded in paraffin. Four micrometer-th ick sections were prepared and immunostained using HLA-A, B and C heavy chains (anti-HLA class 1 ABC antibody W6/32) from DAKO and used at 1:100 dilution. Immunohistochemical staining for PD-L1 used clone SP142 (Spring Bioscience, Berlin, Germany) at 1:200 dilution. Controls were isotype matched. Standard immunostaining was performed as previously described [12]. Briefly, staining was performed with a Dako AutostainerLink 48 instrument (Hamburg, Germany) using an EnVision FLEX/HRP DAB detection kit. All of the sections were scored as positive when the percentage of stained tumor cells in an entire tumor area was greater than 1%.

### 2.7. Preparation of CD8+ Lymphocytes and FACS Analysis

PBMCs and corresponding fresh tumor biopsies were obtained from selected patients (*n* = 4) and processed together, in order to avoid processing-related differences in T cell activation. PBMCs were isolated from buffy coats by density gradient centrifugation of 30 mL of blood collected during surgery. CD8+ T cell were selected from PBMCs by an isolation kit (EasySeptm Direct Human CD8+ T-cell isolation kit, Biolegend, San Diego, CA, USA). The CD8+ T cells were cultured at a concentration of 8 × 10^6^/mL in U multiwells in RPMI-1640, 10% serum medium, expanded with 30 U/mL rIL-2 and stimulated beads™ with CD3/CD28 antibody at 5 μg/mL (Gibco, San Diego, CA, USA) or with 10 µg/mL an isotype control antibody (MOPC-21; Biolegend, Milan, Italy) according to the manufacturer’s instructions. Media were refreshed every three days, including beads and 30 U/mL rIL-2. Then, 100 µL of cultured supernatants for each sample (at 24, 48, and 72 h from activation) and one negative control (CD8 T cells seeded without beads™ CD3/CD28 antibody) were tested for IFNγ levels using a commercial IFN ELISA kit (Thermo Fisher Scientific, Waltham, MA, USA) according to the manufacturer’s instructions. In total, 10^5^ CD8 T lymphocytes from each patient were characterized before and after activation using (BD Multitest™ CD8/CD38/CD3/Anti–HLA-DR BD Catalog No. 340572) mouse monoclonal anti human CD45 (2D1), PE Mouse Anti-Human CD274 (MHI1; BD Biosciences, Franklin Lakes, NJ, USA) and PE Mouse Anti-Human CD279 (EH12.1; BD Bioscience).

FACS analysis of tumor tissue biopsy fragments was performed as follows: Tumor tissue fragments were cultured for 24 h and washed in FACS buffer (PBS, 5 mM EDTA, 1% bovine serum albumin). Then, 10^5^ cells were stained with PE Cy7 Mouse Anti-Human CD90 (5E10) (BD Bioscience) and PE (BD Pharmingen FITC Mouse Anti-Human HLA-ABC, Franklin Lakes, NJ, USA) for 30 min at 4 °C and sorted. Controls were isotype IgG1 and G2 from BD (#342409). All flow cytometry FACS analyses were performed with an ARIA III flow cytometer (BD life Science, San Jose, CA, USA) or BD FACSLyric and analyzed by BD FACSuite (version 1.2).

### 2.8. T Cell Division Cycle Profile by CFSE

CD8+ cells (freshly purified) were labeled with CFSE (Molecular Probes, Eugene, OR). Briefly, cells were washed with serum-free RPMI, (10^7^ cells/mL), labeled with 10 μM CFSE in serum-free RPMI at 37 °C for 10 min and then neutralized with complete RPMI. CFSE-labeled CD8+ T cells were cultured either on anti-CD3 mAb + anti-CD28 mAb-beads (5 μg/mL for each Ab) or alone. CFSE-labeling efficiency after 72 h was detected together, using CD8 antibody staining and a FACScan flow cytometer. Histograms were analyzed using CellQuest software.

### 2.9. Tissue Specimen Cultures

Selected flash frozen tissue specimens (*n* = 4) were thawed, washed in HBSS and processed into ~1 mm^3^ sized aliquots and then distributed into separate wells of a 100 mm dish. Each dish contained one tumor fragment immersed in 10 mL of complete media (RPM I). Plates were incubated at 37 °C with 5% CO_2_. After 1 week of culture, tumor cells from biopsies were assayed for CD90 (mesenchymal antigen) PD-L1 and HLA-I antigens by FACS sorting.

### 2.10. CD8 T Cell Cytotoxicity Assay

Tumor cells from four different biopsies (target cells) were seeded overnight in 3 × 10^3^ cells per well in 96-well plates, then co-cultured with autologous CD8 T cells previously activated (effector cells) at different cell ratios (E/T = 3:1, 10:1 and 20:1). Anti-Human HLA-I (W6/32) DAKO and IgG1 isotype antibodies (MAB002, R&D system, Milan, Italy) at 1 µg/mL were added as controls or HLA-II (CD74 Human AF3590, R&D system).

Following incubation for 24 h at 37 °C and 5% CO_2_, cytotoxicity mediated by MHC-I was measured using a CytoTox-Fluor™ Cytotoxicity kit (Promega, G9261, Milan, Italy). Dose response to Nivolumab was also tested using different concentrations of Nivolumab and IgG1 isotype control. In place of effectors, media alone (spontaneous lysis) or 1% Triton X (maximum lysis) was added to the control wells. The percentage of specific lysis was calculated as follows: % specific lysis = (test sample value) − (spontaneous release value) (maximum release value) − (spontaneous release value) × 100% specific lysis = (test sample value).

### 2.11. Determination of T Cell-Mediated Cell Killing by FACS

A total of 5 × 10^4^ tumor cells from different patient biopsies (n.4) were plated in 24-well flat-bottom plates. Activated CD8+ T cells at ratio, 1:3 (tumor cells: CD8+ T cells) were then added with 1 µg/mL of Nivolumab or 1 µg/mL anti HLA-I antibody (W6/20 DAKO) or isotype control IgG1 (as negative controls) and incubated for 24 h at 37 °C and 5% CO_2_. Cell debris and lymphocytes were removed, and adherent cells were labeled with Annexin V FITC-A antibody and propidium iodide (PI) (ThermoFisher Scientific, Waltham, MA, USA). Nivolumab (Opdivo) MDX-1106 was obtained from Selleckchem.

### 2.12. Statistical Analysis

Data were analyzed by GraphPad Prism version 8. One-way ANOVA were used to compare different groups and two-tailed Student’s *t*-test to analyze intra-group differences. Pearson’s R method was used to test the correlation of patients’ PD-L1 mRNAs with their PD-L1 protein expression (continuous variable). Significant values were indicated as *; *p* < 0.05, **; *p* < 0.01 and ***; *p* < 0.001.

## 3. Results

### 3.1. HLA-I Genotype in Sarcoma Tissues

**Figure 1:**
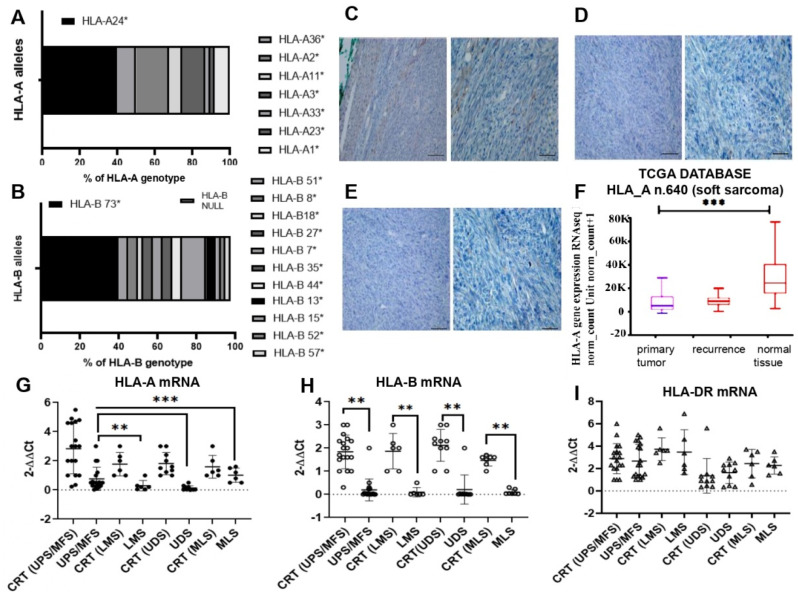
(**A**) HLA-A allele frequency in the sarcoma (STS) population (*n* = 40) determined by Taqman genotyping. (**B**) HLA-B allele frequency in the STS population analyzed by Taqman. (**C**) Representative immunohistochemistry of a low grade myxofibrosarcoma section stained with HLA-I antibody (low magnification on left, higher magnification on right). Moderate brown color indicates positive staining. (**D**,**E**) high grade myxofibrosarcoma tissues negative stained to HLA-I antibody. Scale bars on all images = 100 µm and 50 µm. (**F**) Down-regulation of HLA-A mRNA in soft sarcoma tumors and recurrences assessed in TCGA RNAseq database. (**G**–**I**) Real time PCR dosage of HLA-A (**G**), HLA-B (**H**) and HLA-DR (**I**) mRNAs in various sarcoma tissues, compared to surrounding healthy tissue control (*CRT*). UPS undifferentiated pleomorphic-sarcoma/MFS, undifferentiated myxofibrosarcoma; LMS, leiomyosarcoma; UDS, undifferentiated sarcoma; MLS, myxoid liposarcomas. Relative quantifications are reported as 2^−ΔΔ*Ct*^. ** *p* < 0.01; *** *p* < 0.001.

We studied 40 soft tissue sarcomas (STS) with different histology: Undifferentiated pleomorphic sarcoma (UPS), Myxofibrosarcoma (MFS), Leiomyosarcoma (LMS), Undifferentiated sarcoma (UDS) and Myxoid liposarcoma (MLPS). The main age of patients was 65 years, and 60% were female. Of primary lesions, 20 were grade III and 3 were recurrences (Table 1). Tumor sizes exceeded 5 cm in 45% of patients. Tumors were superficial (46%), deep (29%) or of unknown location. The extremities were the most common primary localization (80%), followed by the trunk (20%). Histological grade III was most common (50%), whereas grade II was found in 12%, grade I in 30% and recurrence in 7%. For primary treatment, most patients underwent surgery (90%), resulting in no residual disease in 82.5%. Additional treatment, such as radiation, was given in neoadjuvant (4%). To investigate the haplotype of HLA I in the sarcoma population, genomic DNA from frozen tumor tissues was genotyped by the multiplex Taqman system. The frequencies of individual allelic groups and haplotypes were determined by exact tests and interactive gene-finder algorithm software. The observed frequencies of HLA class I alleles in our STS population are reported in Supplementary Table S1. HLA-A genotyping analysis uncovered eight different alleles, three of which with a frequency ≥10%. The most commonly observed group of alleles were HLA-A*24, present in 40% of our population, whereas in the Caucasian population, in general, it would be expected to be 20%, as reported by http://www.allelefrequencies.net, accessed on 1 November 2021. Other alleles identified were HLA-A*02 (17,5%), HLA-A*03 (12.5%), HLA-A*36 (10%) and HLA-A*01 (7%) (Figure 1A and Supplementary Table S1). Together, these allelic variants comprised 87% of HLA-A allelic diversity in our sample population. The HLA-B locus is known to be the most polymorphic and variable one, with currently 43 serological identified groups and more than 400 alleles assigned by molecular typing methods (HLA Databank). In our STS population, deletion of the HLA-B region was frequent (10% of cases) mostly in stage III sarcomas. The allelic variant HLA-B*73 was present in 42% of tumors, whereas many other alleles identified were present in low percentages, such as the HLA-B*44 allele (0.45%) (http://www.allelefrequencies.net, accessed on 1 November 2021) (Figure 1B). Our data provide the first description of alleles and haplotype frequencies in STS population and reveal a common allele in class I. Among class II genes, DRB1 exhibited the lowest allelic diversity, and the HLA-DRB1*01 family was most presented (50%). The most frequent haplotype was A*24:10~ ~B*73:01~DRB1*01~present in 50% of myxofibrosarcoma cases (Supplementary Table S1).

### 3.2. Expression of HLA-I Antigens in Sarcoma Tissues

To verify whether qualitative and quantitative differences affect the HLA-I antigen expression, we evaluated their amounts in tumor tissues. The staining with HLA-I antibody of all 40 STS sections indicated a low-intensity signal in grade I and II tumors and none in grade III tumors. Representative cases of myxofibrosarcoma are shown in Figure 1C–E. RNAseq analysis from 640 soft tissue sarcomas listed in the TCGA data base indicated a significant reduction of HLA-A mRNA, compared to normal surrounding tissues (*p* < 0.001, Figure 1F). In our population, we confirmed the data by real time quantification, although in UPS/MFS histology, HLA-A mRNA was higher than in LMS and UDS (Figure 1G). HLA-B mRNA was absent in 10% of cases and lower than control, confirming the genomic data (Figure 1H and Supplementary Table S1). No significant difference was observed for HLA-DR mRNA between control and tumor samples.

### 3.3. PD-L1 Expression in Sarcoma Biopsies

**Figure 2:**
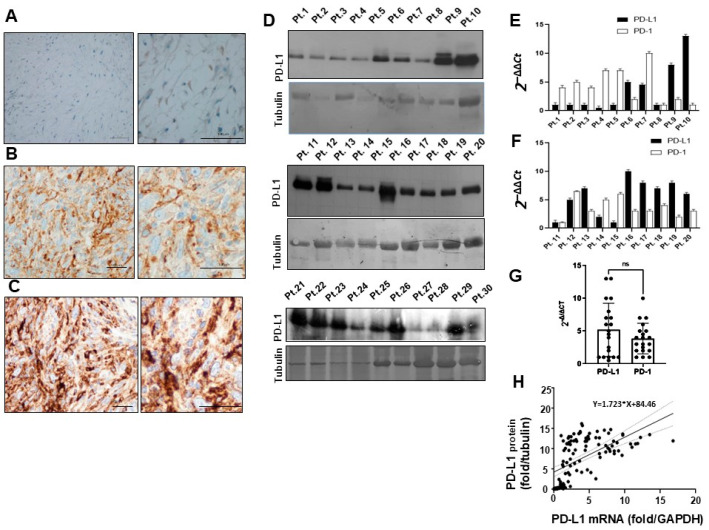
(**A**) Representative negative PD-L1 immunohistochemistry of myxofibrosarcoma at grade I; (**B**) High grade myxofibrosarcoma with moderate staining with anti-PD-L1 antibody. (**C**) High grade myxofibrosarcoma strongly positive to PD-L1 antibody. Low magnification images on the left, high magnification on the right; Scale bars = 200 µm and 50 µm, respectively. (**D**) Western blot of the same amounts of total protein extracts from representative sarcoma tissues, detected with PD-L1 antibody and tubulin control. (**E**,**F**) Representative real-time PCR quantification of PD-L1 and PD-1 mRNA expression in different STS patients (*n* = 20). Relative quantifications were reported as 2^−ΔΔ*Ct*^. (**G**) Quantitative comparison of PD-L1 and PD-1 mRNAs in overall patient population ns (not significant). (**H**) Linear regression between PD-L1 mRNA(fold-change/GAPDH) and protein (fold-change/tubulin) in individual patients (*n* = 40). R^2^ = 0.33, 95% C.I. = 1267 to 2179.

Next, we analyzed the relationship between HLA-I and PD-L1 mRNAs and protein expression. PD-L1 immunohistochemistry indicated that the positive cells were >5%, mainly in grade III tumors, negative for HLA-I antibody, and in some sporadic cases of grade I (Figure 2A–C and Supplementary Table S2). Altogether, immunohistochemistry data indicated that an HLA-I positive signal was generally associated with low grade tumor and PD-L1 to high grade tumor (Supplementary Table S2). In contrast, Western blots detected PD-L1 protein in the almost all tissues (Figure 2D, Supplementary Figure S1). In individual biopsies, PD-L1 and PD-1 mRNAs were well-balanced and expressed five times higher than in HLA-A mRNAs (Figure 2E–G). Additionally, a significant correlation between intracellular PD-L1 protein (reported as fold change versus γ tubulin) and PD-L1 mRNAs (fold change versus GAPDH) was observed (C.I. ranging from 1267 to 2179) (Figure 2H), suggesting that an intracellular exporting mechanism affects the cell surface expression of PD-L1.

### 3.4. Patient’s T Cells Characterization

**Figure 3:**
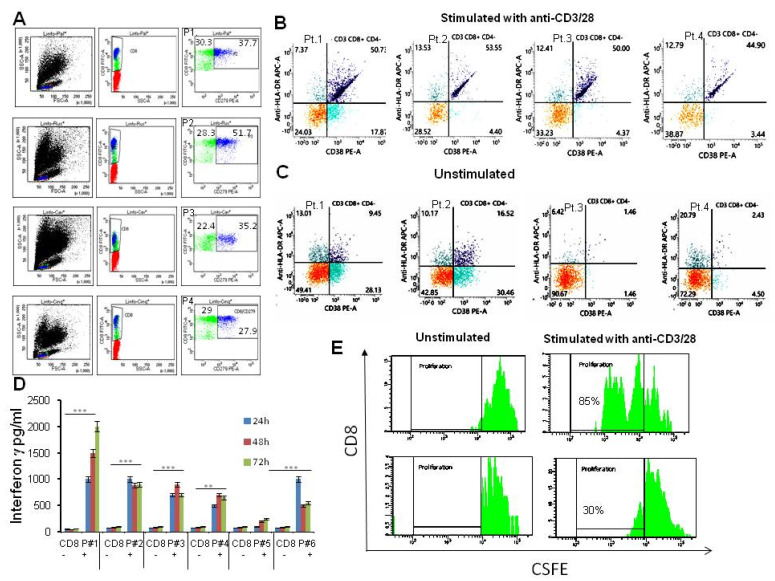
Flow gate plot quantification of patients’ CD8 populations. (**A**) PBMCs from four STS patients (Pt.1, Pt.2, Pt.3 and Pt.4) sorted for CD8 (green dots) and PD-1 antigens (blue dots). Their percentage is indicated in individual quadrants. (**B**,**C**) Flow gate plot quantification of CD38 and HLA-DR positive antigens in the CD3+/CD8+/CD4− cell population stimulated for 24 h with CD3/CD28 beads (**B**) or unstimulated (**C**). (**D**) ELISA quantification of interferon gamma (IFNγ) production in response to stimulation of CD8+ cells isolated from 6 randomly chosen patients. CD8+ cells were added in triplicates to U-bottom cell culture plates containing CD3/CD28 beads in cell culture medium (+) or medium alone (−) and cultured at 37 °C under 5% CO_2_ for different times, as indicated. After the incubation, the culture supernatant was collected, and IFNγ was detected by ELISA. Optical density (OD) values from the spectrophotometer at 450 nm were used to construct a standard curve and quantify IFNγ expression. Data are means ± SD of three independent experiments. ** *p* < 0.01, *** *p* < 0.001. (**E**) rIL-2 stimulated proliferation of patient CD8+ cells quantified by CFSE. CD8+ cells were stained with 5 μM CFSE at a concentration of 10^7^ cells/mL and stimulated with rIL-2 (30 U/mL) or medium for 72 h. Cells were collected and stained with mouse anti-human CD8 antibodies. Samples were assayed by flow cytometry and data were analyzed using FlowJo software. Representative proliferation graphs of the unstimulated and stimulated CD8+ T cells from two patients at 72 h are shown.

We then asked whether low tumor HLA-I expression may affect the recognition and killing by autologous T cells. To this aim, CD8+ T cell subpopulations were selected from patients’ peripheral blood mononuclear cells (PBMCs). The baseline flow cytometry for the CD8 and PD-1 antigens indicated that high proportions (average 30–50%) of CD8+ T cells were also positive for PD-1, suggesting that these had adapted an immune suppressing mechanism upon exposure to the tumor microenvironment (Figure 3A). Moreover, the basal activation markers (CD38 and HLA-DR) ranged between 2–10% of cells (Figure 3C). Patient’s CD8+ T cells after stimulation with anti-CD3/CD28 beads for 24 h showed a variable spectrum of activation markers. In particular, CD3+ CD8+ CD4− cells expressed CD38 and HLA-DR antigens in a percentage ranging between 35 to 90% (Figure 3B,C). All stimulated CD8+ T cell populations generated a variable amount of IFNγ assessed by ELISA, compared to unstimulated controls. This was sustained for up to 72 h (Figure 3D). To assess whether cell proliferation, as an index of vitality, occurred during the stimulation, patient’s CD8+ T cells, were monitored for number of cell divisions using the division-tracking dye carboxyfluorescein diacetate succinimidyl ester (CFSE). After 72 h of stimulation with rIL2, CD8+ cells from representative patients showed an increase of the proliferation rate ranging between 30–85%, compared to unstimulated cells (Figure 3E).

### 3.5. Characterization of HLA-A24* and PD-L1 Antigens in Selected Biopsies

**Figure 4:**
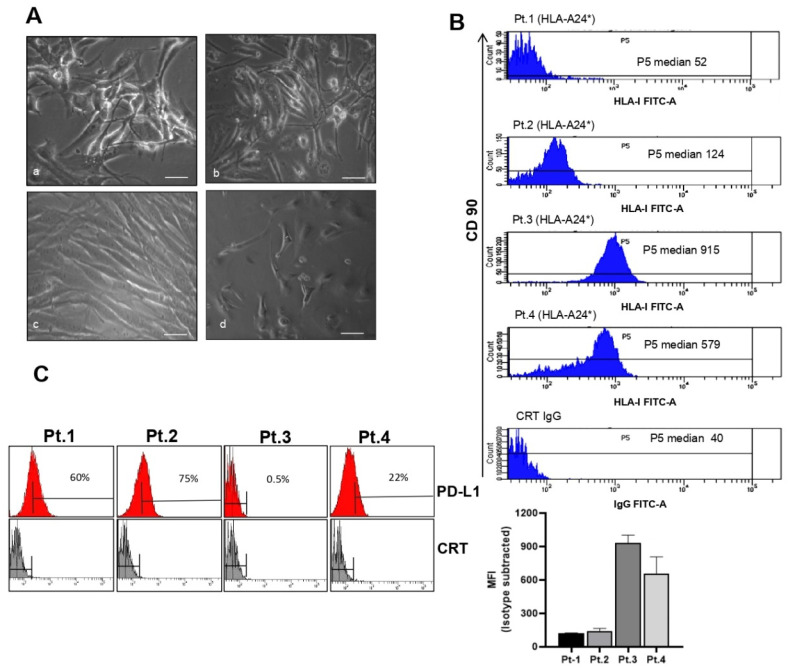
(**A**) Representative image of four primary cell cultures obtained from fresh sarcoma tissue sections with different histology: myxofibrosarcoma (**a**), pleomorphic sarcoma (**b**), undifferentiated sarcoma (**c**) and undifferentiated sarcoma (**d**); Scale bars = 100 µm. (**B**) Surface HLA-I expression determined by flow cytometry of biopsies from different patients using anti-HLA-I mouse monoclonal antibody FITC-A and anti-CD90 (mesenchymal antigens). Median of fluorescence is reported for the same 4 patients and a negative control (polyspecific IgG). The bar graph reports the median of fluorescence intensity (MFI) with monoclonal HLA-I -FITC-A minus MFI isotype control normalized for 1 × 10^4^ FACS events. Error bars represent SEMs of 3 measurements in at least two independent experiments. Pt.1 indicates patient biopsy number 1, Pt.2 indicates patient biopsy number 2, Pt.3 indicates patient biopsy number 3, Pt.4 indicates patient biopsy number 4. (**C**) Cells from biopsies analyzed for PD-L1 expression by FACS. The bar indicates the percentage of positive cells.

To verify whether HLA-A24* restricts CD8+ T cells response, we assessed the expression level of antigen HLA-A24* in tumor biopsies. To this aim, STS tissues fresh after surgery from grade III sarcomas patients 1 (Pt.1) and 2 (Pt.2), and grade I patients 3 (Pt.3) and 4, (Pt.4) genotyped for the HLA-A24* allele, were fragmented and cultured for 24 h (Figure 4A). The growing cells from these sources were then sorted for HLA-A, PD-L1 antigens and mesenchymal antigen CD90, and reported as the population’s fluorescence intensity subtracted from that of controls (Figure 4B,C). In representative patients with different STS histology (Figure 4A), CD90 antigen recognized most of the tumor cells (Figure 4B). The median fluorescence of HLA-I intensity (MFI) ranged from 52 to 915, indicating that functional epitopes necessary for CD8 binding were expressed at different level in fresh biopsies. In the same patients, PD-L1 antigen was positive in 25–75% of cells (Figure 4C). Specifically, Pt.1 grade III myxofibrosarcoma and Pt.2 undifferentiated sarcoma showed the lowest HLA-I (MFI = 52 and 124) and positivity for PD-L1 in 60 and 75% of cells, respectively, whereas grade I Pt.3 and Pt.4 showed the highest expression of HLA-I (MFI = 915 and 579) and positivity for PD-L1 in 0.5 to 22% of cells, respectively.

### 3.6. Immune Sensitivity of HLA-A24* Tumors to Cytotoxicity Mediated by TCR

**Figure 5:**
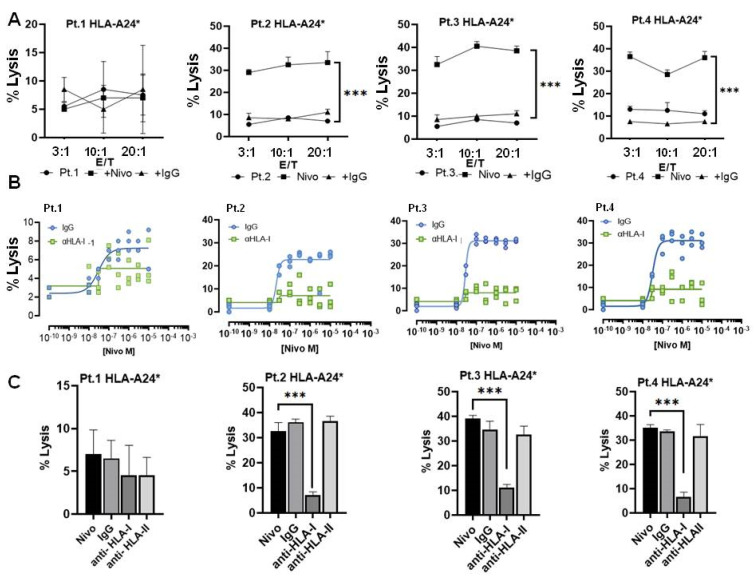
(**A**) Cytotoxicity assay of autologous CD8+ T cells stimulated with CD3/CD8 antibodies for 24 h. Stimulated CD8 T cells were then tested for their cytotoxicity toward HLA-A24* autologous sarcoma tumor cells using different effector/target cell ratios (E/T) in four patients (Pt.1, Pt.2, Pt.3 and Pt.4) with different histology (see legend of Figure 4A). Nivolumab or IgG1 isotype antibodies at 1 µg/mL were added as adjuvant effector or control. Following incubation for 24 h, cytotoxicity was measured using a CytoTox-Fluor™ Cytotoxicity kit (Promega, G9261). (**B**) Dose response to Nivolumab in a cytotoxity assay of autologous stimulated CD8+ T cells and target cells from patients Pt.1, Pt.2, Pt.3, Pt.4 (at a ratio 3:1). Different concentrations of Nivolumab were incubated for 24 h together with IgG isotype control or anti-HLA-I antibodies blocking MHC-I (at 1 µg/mL) and the percent of cell lysis was determined by CytoTox-Fluor™. (**C**) Cytotoxicity inhibition assay evaluating TCR/HLA-I engagement. Stimulated CD8+ T cells incubated with autologous patient tumor cells positive for allele HLA-A24* in the presence of Nivolumab or indicated monoclonal antibody. Values for percentage of specific lysis were calculated as follows: % specific lysis = (test sample value) − (spontaneous release value)(maximum release value) − (spontaneous release value) × 100% specific lysis = (test sample value). Results reported the mean of three independent experiments ± SD. *** *p* < 0.001.

To evaluate the immune sensitivity of HLA-A24* tumors to cytotoxicity mediated by autologous CD8+ T cells, tumor cells from fresh biopsies with different histology were co-cultured with autologous CD8+ T cells. CD8+ cells pre-activated for 24 h were incubated at different ratios between effectors (CD8+ T cells) and target (tumor) cells, both with or without Nivolumab, and their cytolytic activity assayed by CytoTox-Fluor™. As indicated in Figure 5A, all effector/target cell ratios resulted in low cytolysis with a maximum around of 10% lysed cells in patients 3 and 4 with higher expression of HLA-I. An increase of CD8+ cells lytic activity was observed by addition of Nivolumab, blocking PD-1/PD-L1 recognition (Figure 5A) in a dose-dependent manner (Figure 5B), with individual variability in the response. In patient 1 that showed the lowest HLA-I and 60% of positive cells to PD-L1, the addition of Nivolumab did not increase lysis mediated by autologous CD8+ T cells. Then, we attempted to determine whether CD8+ T cell-mediated tumor lyses occurs via the TCR-dependent mechanism. For this purpose, CD8+ T cells were co-cultured with tumor cells from biopsies (at a 3:1 ratio between effector and target cells) in the presence of selected concentrations of Nivolumab. As shown in Figure 5C, CD8+ cell-mediated tumor lyses decreased by the addition of anti-HLA class I mAb (W6/32), but not by the addition of either an anti-HLA class II (HLA-DR) mAb or an isotype control. Again in patient 1, no significant difference was observed. These results suggested that the cytotoxicity of stimulated CD8+ T cells is restricted by low HLA-I expression in cancer cells and the TCR blockade inhibited CD8 killing tumor cells.

### 3.7. Reactivity of T Cell against HLA-A24 Restricted Antigen

**Figure 6:**
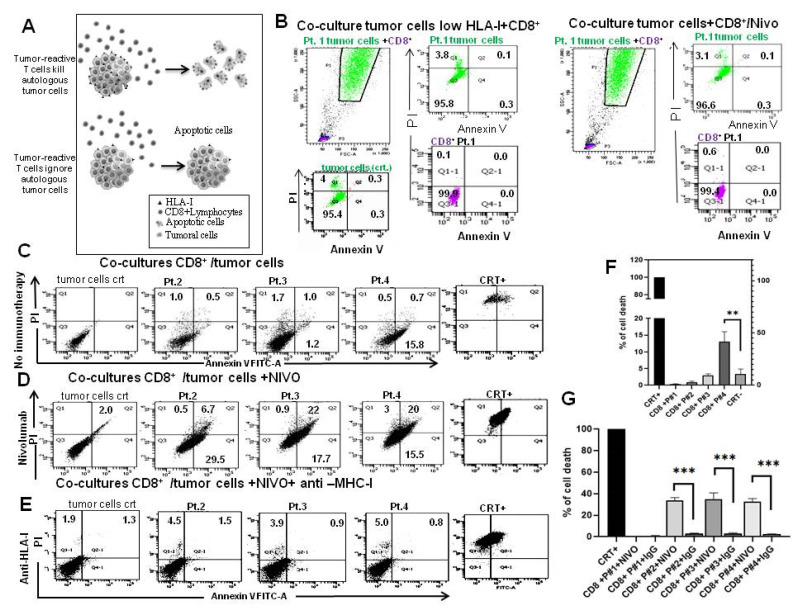
(**A**) To determine the ability of CD8+ T cells to kill autologous tumor cells depends on the expression of substantial HLA24* antigen; therefore, we used the following assay: Primary cell cultures were established from individual biopsies and then co-cultured with CD8+ T cells obtained from blood of the same patient. CD8+ T cells from 4 patients were stimulated for 24 h with CD3/CD28 beads. The cytotoxic effect of CD8+ T cells was then evaluated by co-cultures with autologous tumor cells, using flow cytometry. (**B**) Flow gate plot of co-cultures of CD8+ T cells with tumor cells from the same patient (Pt.1) with low HLA-I expression level (MFI = 52). CD8+ T cells (pink) and tumor cells (green) were gated separately on the base of FSC and then plotted for Propidium iodide (PI) and Annexin V FITC-A in the absence (left group) or presence (right group) of 1 µg/mL Nivolumab. Percentages of positive cells are indicated in the quadrants. (**C**,**D**) Flow gate plot by PI and Annexin V of co-cultures of CD8+ T cells and tumor cells of patients 2, 3 and 4 (Pt.2, Pt.3 and Pt.4) in the absence (**C**) or presence of 1 µg/mL Nivolumab (**D**). (**E**) The ability of autologous CD8+ T cells to kill tumor cells was assessed by PI and Annexin V positive cells in presence of HLA-I antibody. (**F**,**G**) Bar graphs reporting the percentage of cell death in co-cultures with autologous CD8+ T cells from patients in the absence (**F**) or presence of Nivolumab and IgG isotype control (**F**). Error bars represent SD of three replicates. ** *p* < 0.001, *** *p* < 0.001.

We further examined whether the cytotoxic activity of CD8+ T cells against autologous cancer cells can be used to assess the efficiency of tumor destruction. To determine this, the same cell samples investigated above were co-cultured with autologous tumor reactive T cells for 24 h and cell death was visualized by FACS. Results were then differentiated by the patients’ HLA-I. The mean fluorescence intensity (MFI) was 52 for Pt.1; 124 for Pt.2, 915 for Pt.3 and 579 for Pt.4. Tumor cells expressing low HLA-I (MFI = 52) and positive to PD-L1 (60% of positive cells) were not killed by autologous activated T cells, not even in the presence of Nivolumab (Figure 6A). The highest cell death (16%) due to effect of activated CD8+ T cells was observed in Pt.3 with MFI = 915 and 0.5% of cells positive to PD-L1 (Figure 6B,F). In this patient, the addition of Nivolumab increased the cell death (40%) (Figure 6B,C,G). In Pt.4 with HLA-I antigens (MFI = 574) and positive for 22% to PD-L1, the capability of CD8+ cells to kill tumor cells was very low (Figure 6B,C,F), whereas the addition of Nivolumab increased it to 38% (Figure 6B,C,G). The addition of HLA-I blocking antibody abolished tumor cells death and demonstrating antigen specific CD8+ T cell response (Figure 6D,G). All together, these data indicate that the CD8+ T cells stimulated in vitro can exert cytotoxicity against cancer cells, and that their cytotoxic activity is dependent in part on TCR recognition of HLA-I. However, the increase in T cell cytotoxicity is not linearily dependent by HLA-I expression but it is capped at 40% in presence of Nivolumab. Representative adherent tumor cells with higher HLA-I expression were also incubated with polyspecific HLA-I, IgG serum and complement factors in Terasaki lymphocyte-toxicity tests. The addition of complement factors did not induce cell death in any tumor cells tested, independently of HLA-I expression levels (Supplementary Figure S2), suggesting that tumor cells were resistant to complement-mediated killing.

## 4. Discussion

The present study characterizes HLA-I genetic variation in sarcoma tissues and optimizes a test that permits the evaluation of autologous cytotoxic T cell-mediated tumor destruction in individual patients. It has previously been established that cytotoxic T cells mediate the recognition and death of tumor cells in a patient-specific manner [32]. Models to measure T cells tumor interaction are therefore of great interest for implementation of personalized medicine, but have not been reported for sarcomas [12,13,33].

Two main aspects that influence the intrinsic capacity of T cells to recognize human cancer tissues are: the genetic diversity of human leukocyte antigens (HLA-I) and neoantigens expressed [33]. We genotyped 40 soft tissue sarcomas for human leukocyte class I (HLA-I)—A and B and class II HLA-DR. Results identified a common haplotype HLA-A 24* and HLA-B73* that was strongly down-regulated compared to healthy tissues, and a genetic deletion of the HLA-B locus in some myxoid liposarcoma (MLPS). HLA-I molecules are critical for antigen presentation to CD8+ cytotoxic T-lymphocytes [27] and the HLA*24 allele expressed in other cancer types was also reported to escape T lymphocytes response [33]. If tumors do not express MHC class I, cytotoxic T cells cannot target them and therefore therapeutic immune check-point antibodies may not be effective. In soft tissue sarcomas, both immunostimulatory and targeted immunotherapy is of great interest. The use of T cells recognizing specific antigens, such as New York esophageal squamous cell carcinoma 1, presented by HLA A02* (NY-ESO-1), was shown to be safe and effective in metastatic synovial sarcoma or myxoid liposarcoma [34,35,36,37]. Trials that have already investigated immune checkpoint therapy targeting PD-1/PD-L1 for some sarcoma histotypes pose a challenges to the identification of subtype specific genetic and molecular alterations [38].

We now established that sarcoma select rare alleles of HLA-I loci and/or delete HLA-B locus and strongly down-regulate HLA-I mRNAs compared to normal tissues, consistent with similar findings reported for other cancer types [39]. These events cause MHC class I to be often undetectable by immunohistochemistry and CD8 T cells cannot bind it.

Our study links MHC class I expression, immunotherapy targeting PD-L1 and individualized cancer therapy. We reported a cytoplasmic accumulation of PD-L1 protein in sarcoma tissues, which correlated with PD-L1 mRNAs, even though in most low-grade cases, immunohistochemistry did not detect PD-L1 in tumor sections. Conventional histological detection of PD-L1 is an important determinant of patient eligibility for immunotherapy and to predict its clinical efficacy [2,12]. Our data suggest that only a small percentage of PD-L1 protein expressed is exported to the cell surface or recognized by diagnostic antibodies [29,30]. We have now demonstrated the relationship between rare HLA-I alleles and PD-L1 by functional CD8+ T cell cytotoxicity tests. This interaction raises intriguing questions and provides new insights into mechanisms of current immunotherapies [40,41].

Moreover, to assess the possibility to evaluate tumor sensitivity to immunotherapy for individual patients, we adopted a specific T cells test. We did so by establishing primary cell cultures from some fresh sarcoma tissues and expanded autologous CD8+ T cells from peripheral blood. The ability to expand reactive circulating tumor CD8+ T cells provides a strategy for the generation of patient specific T cells by bypassing the necessity to isolate tumor-infiltrating lymphocytes [33]. In parallel, we used co-cultures of tumor and CD8+ T cells in the presence or absence of immunotherapeutic drugs of interest, to identify candidate responders to immunotherapy. Our data suggest that tumors can successfully evade CD8+ T cell-mediated cytotoxicity by down-regulating or deleting expression of HLA-I alleles. At the same time, results indicated the potential of such tests to predict individual responses to immunotherapy. Obviously, further studies with larger patient numbers will be needed to establish the relative contribution of the HLA-I-dependent T cell killing of tumor cells and the predictive power of such assays.

Another important finding of our study is the identification of a common haplotype in soft sarcomas. Epitope discovery is an essential step in the design of novel immunotherapy. Recent reports indicate that selected tumor epitopes can be used to educate primary T cells to specific recognition of cognate peptide-HLA complex, efficiently kill target cells and open new prospective immune therapies [40]. Our finding that a rare haplotype is common to sarcoma may indicate that evaluation of novel targets for bi-specific engagers and TCR therapies could be considered.

## 5. Conclusions

The major histocompatibility complex plays a key role in the clinical outcome of cancer immunotherapy, including treatment with antibodies blocking immune checkpoint molecules. Our study genotyped a heterogenous group of soft tissue sarcomas for HLA-I. With the help of the Taqman realtime method, we identified a common haplotype and astrong down-regulation of HLA-I expression. Although the tumor microenvironment is very complex, we described and tested an assay using individual patients’ CD8+ T cells in the presence of immunotherapeutics. Such an assay could be useful to identify patients who may benefit from immunotherapy.

## Figures and Tables

**Table 1:** Characteristics of patients enrolled in the study.

Patients	40
**Diagnosis**	
*Myxoid liposarcoma (MLPS)*	6
*Undifferentiated sarcoma (UDS)*	10
*Undifferentiated Pleomorphic sarcoma (UPS)*	6
*Leiomyosarcoma (LMS)*	6
*Myxofibrosarcoma (MFS)*	12
**Age** yrs	65 ± 1
**Gender**	
*Male*	16
*Female*	24
**Localization**	
*Upper extremities*	12
*Lower extremities*	20
*Trunk*	8
***Stage (AJCC system***)	
I	12
II	5
III	20
*Recurrence*	3
***Surgery***	40
***Radiotherapy***	
*No*	36
*Adjuvant*	4
***Tumor size***	
≥5 cm	18
≤5 cm	22
***Residual disease after surgery***	
*R0*	33
*R1/R2*	7

## Data Availability

The datasets [GENERATED] for this study can be found in the supplementary materials.

## Supplementary Materials

The following supporting information can be downloaded at: https://www.mdpi.com/article/10.3390/cancers14143414/s1, Figure S1: Terasaki assay. Figure S2: Uncropped Western blot of patient tissues. Table S1: frequencies of HLA class I alleles in our STS population. Table S2: Expression of PD-L1 and HLA-1 in sarcoma population. Table S3: qRT-PCR primer sequences.
