# Correction: Mosca et al. Sarcoma Common MHC-I Haplotype Restricts Tumor-Specific CD8+ T Cell Response. *Cancers* 2022, *14*, 3414

**DOI:** 10.3390/cancers18020276

**Published:** 2026-01-16

**Authors:** Laura Mosca, Alessandra de Angelis, Andrea Ronchi, Annarosaria De Chiara, Flavio Fazioli, Carlo Ruosi, Lucia Altucci, Mariarosaria Conte, Filomena de Nigris

**Affiliations:** 1 Department of Precision Medicine, School of Medicine, University of Campania “Luigi Vanvitelli”, 80138 Naples, Italy; 2 Pathology Unit, Department of Mental and Physical Health and Preventive Medicine, University of Campania “Luigi Vanvitelli”, 80138 Naples, Italy; 3 Division of Anatomy, Istituto Nazionale Tumori IRCCS—Fondazione G. Pascale, 80131 Naples, Italy; 4 Division of Skeletal Muscle Oncology Surgery, Istituto Nazionale Tumori IRCCS—Fondazione G. Pascale, 80131 Naples, Italy; 5 Department of Public Health, School of Medicine, University Federico II, 80131 Naples, Italy; 6 BIOGEM, Molecular Biology and Genetics Research Institute, 83031 Ariano Irpino, Italy

## Error in Figure 2A

In the original publication [1], there were mistakes in Figure 2A as published. Figure 2A was an overlapping photo taken from the histology of the same patient as shown in Figure 1E, instead of the patient indicated in the legend. Since the correct images of both patients are negative controls devoid of specific staining, the error was not immediately detected during figure assembly or revision. The corrected Figure 2A appears below. The authors state that the scientific conclusions are unaffected. This correction was approved by the Academic Editor. The original publication has also been updated.

**Figure 2:**
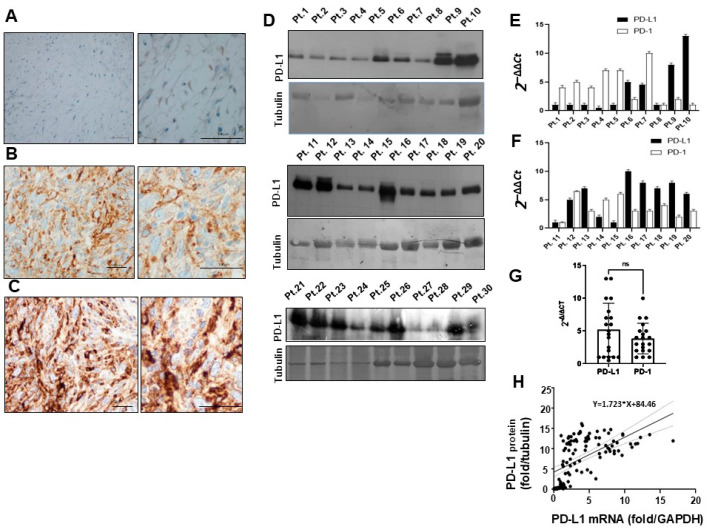
(**A**) Representative negative PD-L1 immunohistochemistry of myxofibrosarcoma at grade I; (**B**) High grade myxofibrosarcoma with moderate staining with anti-PD-L1 antibody. (**C**) High grade myxofibrosarcoma strongly positive to PD-L1 antibody. Low magnification images on the left, high magnification on the right; Scale bars = 200 µm and 50 µm, respectively. (**D**) Western blot of the same amounts of total protein extracts from representative sarcoma tissues, detected with PD-L1 antibody and tubulin control. (**E**,**F**) Representative real-time PCR quantification of PD-L1 and PD-1 mRNA expression in different STS patients (*n* = 20). Relative quantifications were reported as 2^−∆∆*Ct*^. (**G**) Quantitative comparison of PD-L1 and PD-1 mRNAs in overall patient population ns (not significant). (**H**) Linear regression between PD-L1 mRNA(fold-change/GAPDH) and protein (fold-change/tubulin) in individual patients (*n* = 40). R^2^ = 0.33, 95% C.I. = 1267 to 2179.
