# Supplementary material for: Sarcoma Common MHC-I Haplotype Restricts Tumor-Specific CD8+ T Cell Response
Source: Cancers (Basel). 2022 Jul 14;14(14):3414. doi: 10.3390/cancers14143414 (PMC9322060; doi:10.3390/cancers14143414)
Supplement: Supplementary file 1 [file cancers-14-03414-s001.zip › cancers-1793650-supplementary.pdf]

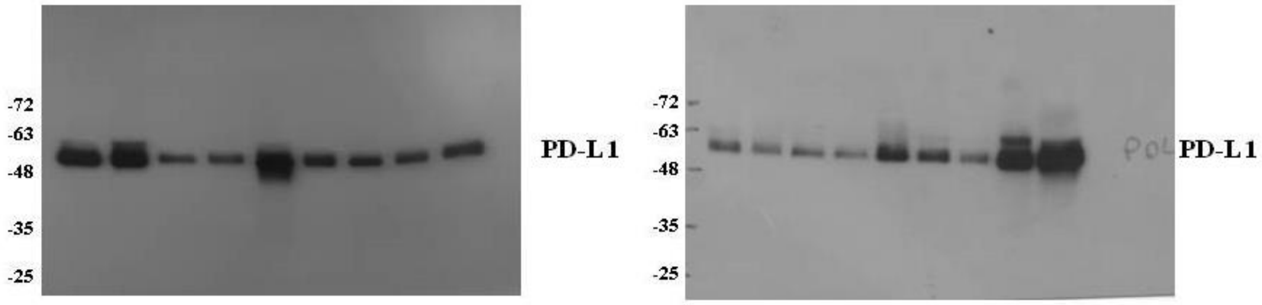

Figure S1. Uncropped western blot of protein extracts from sarcoma patients PD-L1 antibody.

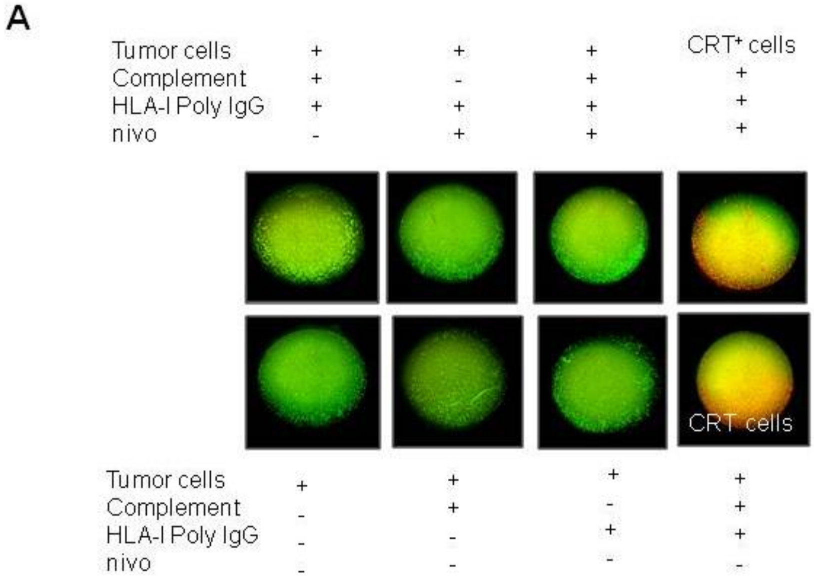

Figure S2. Terasaki cytotoxic assay. Tumor cells from patient 2 (MFI: 916) were plated at a density of 1 × 10^3 cells/well and treated without or with polyspecific HLA-1IgG, complement, and 1μg/mL nivolumab, as indicated. Positive control were 10^3 lymphocytes incubated with HLA-1IgG and cimplement.

Table S1. HLA-A, B and DRB1 haplotypes in surgery sections from sarcoma patients. The frequency refers to caucasic population.

Genotype	*Frequency*	*Genotype*	*Frequency*	*Genotype*	*Frequency*
**HLA-A**		**HLA-B**		**HLA-DRB1**	
HLA-A*1:23/A*24:10	0.0	HLA-B*73:01	0.0	DRB1*01:01/DRB1*08:01	5
HLA-A*24:10/	0.0	HLA-B*73:01	0.0	DRB1*01:01/DRB1*08:01	
HLA-A*36:04	0.0	HLA-B*73:01/B*40:94	0.0	DRB1*01:01/DRB1*08:01	
HLA-A*24:01/A*1:23,	0.0	HLA-B*73:01/B*40:94	0.0	DRB1*01:01/DRB1*08:01	2
HLA-A*24:10/A*24:46	0.0	HLA-B*73:01/B*40:94	0.0	DRB1*01:01/DRB1*08:01	2
HLA-A*33:01/A*66:03	7.7	null		DRB1*01:01/DRB1*08:01	3
HLA-A*36:01/A*66:03	0.0	null		DRB1*01:01/DRB1*08:01	3
HLA-A*24:10/A*24:46	0.0	null		DRB1*08:01	4
HLA-A*24:10/A*24:46	0.0	null		DRB1*08:01	4
HLA-A*24:10/A*24:46	0.0	HLA-B*73:01/B*40:94	0.0	DRB1*01:01/DRB1*08:01	6
HLA-A*24:10/A*24:46	0.0	HLA-B*73:01/B*40:94	0.0	DRB1*01:01/DRB1*08:01	6
HLA-A*24:10/A*1:23	0.0	HLA-B*73:01/B*40:40	0.0	DRB1*01:01/DRB1*08:01	7
HLA-A*24:10/A*1:23	0.0	HLA-B*73:01/B*40:40	0.0	DRB1*01:01/DRB1*08:01	7
HLA-A*24:10	0.0	HLA-B*73:01/B*40:40	0.0	DRB1*01:01/DRB1*08:01	8
HLA-A*24:10/A*1:23	0.0	null	0.0	DRB1*01:01/DRB1*08:01	8
HLA-A*36:04	0.0	HLA-B* 73:01	0.0	DRB1*01:01/DRB1*08:01	9
HLA-A*24:10	0.0	HLA-B* 73:01	0.0	DRB1*01:01/DRB1*08:01	10
HLA-A*01:01	26.9	HLA-B*08:01	9.9	DRB1*03:01	1
HLA-A*02:01	53.8	HLA-B*18:01	0.0	DRB1*11:04	2
HLA-A*02:01	53.8	HLA-B*27:02	0.0	DRB1*16:01	3
HLA-A*03:01	19.2	HLA-B*07:02	13.3	DRB1*16:01	3
HLA-A*11:01	15.4	HLA-B*35:01	26.7	DRB1*16:01	
HLA-A*02:01	53.8	HLA-B*13:02	0.0	DRB1*07:01:	2
HLA-A*02:01	53.8	HLA-B*44:27	0.0	DRB1*16:01	2
HLA-A*02:01	53.8	HLA-B*52:01	6.7	DRB1*15:02	2
HLA-A*11:01	15.4	HLA-B*35:01	26.7	DRB1*01:01	
HLA-A*24:02	11.5	HLA-B*13:02	5.3	DRB1*07:01	0
HLA-A*02:01	53.8	HLA-B*35:03	0.0	DRB1*16:01	2
HLA-A*02:01	53.8	HLA-B*57:01	7.0	DRB1*07:01	2
HLA-A*03:01	19.2	HLA-B*08:01	9.9	DRB1*15:01	3
HLA-A*03:01	19.2	HLA-B*15:01	3.6	DRB1*15:02	3
Genotype		*Genotype*		*Genotype*	*Frequency*
**HLA-A**		**HLA-B**		**HLA-DRB1**	
HLA-A*03:01	19.2	HLA-B*35:0	0.0	DRB1*04:03	3
HLA-A*03:01	19.2	HLA-B*44:27	0.0	DRB1*16:01	3
HLA-A*11:01	15.4	HLA-B*52:01	0.0	DRB1*14:54	
HLA-A*23:01	11.5	HLA-B*44:03	0.0	DRB1*07:01	
HLA-A*24:02	11.5	HLA-B*07:02	0.0	DRB1*15:01	0
HLA-A*24:02	11.5	HLA-B*51:01	0.0	DRB1*16:01	0
HLA-A*1:23	0.0	HLA-B*73:01	0.0	DRB1*01:01/DRB1*08:01	1
HLA-A*1:23	0.0	HLA-B*73:01	0.0	DRB1*01:01/DRB1*08:01	1
HLA-A*24:10/A*24:46	0.0	HLA-B*73:01/B*40:94	0.0	DRB1*01:01/DRB1*08:01	
HLA-A*36:04	0.0	HLA-B*73:01/B*40:94	0.0	DRB1*01:01/DRB1*08:01	0

Table S2. Expression of PD-L1and HLA-1 in sarcoma population.

	Grade I	*Grade II*	*Grade III + recurrence*
Cases (n)	12	5	23
HLA-A24*	6	2	8
HLA-B73*	9	1	7
Anti-PD-L1 (> %5 positive cells)	1	2	7
Anti-HLA-ABC (> %5 positive cells)	4		

Table S3. qRT-PCR primer sequences.

	**Gene Forward primer (5′-3′)**	**Reverse primer (5′-3′)**
HLA-A	GATTACATCGCCTTGAACGAGG	AGAGACAGCGTGGTGAGTCAT
HLA-B	CAGTTCGTGAGGTTCGACAG	CAGCCGTACATGCTCTGGA
PD-L1	GGACAAGCAGTGACCATCAAG	CCCAGAATTACCAAGTGAGTCCT
HLA-DRA	TCTGGCGGCTTGAAGAATTTG	GGTGATCGGAGTATAGTTGGAGC
PD-1	GCTCCAAAGGACTTGTACGTG	TGATCTGAAGGGCAGCATTTC
GAPDH	CCCCCGGTTTCTATAAATTGAGC	AAGAAGATGCGGCTGACTGT
